# Comparison of the Chemical Composition and Pharmacological Effects of the Aqueous and Ethanolic Extracts from a Tibetan “Snow Lotus” (*Saussurea laniceps*) Herb 

**DOI:** 10.3390/molecules17067183

**Published:** 2012-06-12

**Authors:** Tao Yi, Hongwing Lo, Zhongzhen Zhao, Zhiling Yu, Zhijun Yang, Hubiao Chen

**Affiliations:** School of Chinese Medicine, Hong Kong Baptist University, Hong Kong, China

**Keywords:** *Saussurea laniceps*, transesterification, anti-inflammatory, anti-nociceptive, anti-oxidant

## Abstract

To understand the impacts of different processing methods on the composition and effects of the herb *Saussurea laniceps* (SL), the present study report the first comparison of the chemical constituents of aqueous and ethanolic SL extracts using chromatographic analysis, and to compare their pharmacological effects in a mouse anti-inflammatory, anti-nociceptive model and an *in vitro* anti-oxidant test. Chemical comparison demonstrated that the types of chemicals in the two extracts were identical, but the contents of the main constituents in the aqueous extract were lower than those of the ethanolic extract. A transesterification of dicaffeoylquinic acids took place in the aqueous extract during boiling. As for pharmacological effects, oral administration of aqueous and ethanolic SL extracts significantly inhibited croton oil-induced mice ear edema, and significantly inhibited acetic acid-induced mice writhings, respectively. In the DPPH anti-oxidant activity test, the IC_50_ values were calculated as 409.6 mg/L and 523.4 mg/L for the ethanolic and aqueous extracts, respectively. The inhibitory effects of the ethanolic extract were more potent than those of the aqueous extract in all pharmacological tests, although there was no significant difference. This study suggests that the two preparations should be distinguished when used.

## 1. Introduction

Process-induced changes in herbs, which can influence their chemical composition and even the overall attributes of a medicine, is an important research direction of medicinal study [1]. Herbal medicine is a flourishing field in medical science due to the increasing health-consciousness of patients. However, many of these herbs are used according to traditional beliefs, without any scientific verification of their efficacy [2,3]. Thus, the chemical and pharmacological aspects of herbs have attracted more attention, particularly when their composition and efficacy may be affected by different processing conditions.

“Snow Lotus” is a well-known herbal medicine in China, widely prescribed for the treatment of rheumatoid arthritis, stomach ache and dysmenorrhea [4]. “Snow Lotus” herb is derived from several species of the *Saussurea* genus in the Compositae family [5,6]. In our previous study, *S. laniceps* (SL) exerted more potent effects than other species against experimental edema and pain in animal models [7]. However, SL was traditionally processed by steeping in spirit (ca. 40–60% ethanol), but nowadays is processed by boiling in water [8]. In clinical practice, these two preparations of SL, including tincture and decoction, are equally prescribed for the treatment of the same diseases, although no study has been done on the relative efficacy of the two SL preparations [4,5,6]. Therefore, there are two unresolved problems in the current application of SL: (1) whether they are identical in chemical composition; and (2) whether they are equivalent in efficacy. A comparative study of the two processing methods is important because, firstly, safety and efficacy are key requisites for herbs [9], and evidence-based efficacy associated with consumption of the herbs may increase patient confidence [10]. Secondly, this comparison has a special significance for alcoholics and other people who may prefer not to take alcoholic beverages. These people cannot consume tinctures, but they may still be eager to enjoy the health benefits derived from SL, and water decoction offers that option. However, these studies have not been reported so far. Therefore, to encourage safe and reasonable use of the two related preparations, a comparison of their chemical composition and pharmacological activities is needed.

In the present study, we compared the chemical composition of SL aqueous and ethanolic extracts processed by boiling in water and by steeping in spirit containing 50% ethanol. Moreover, we evaluated the anti-inflammatory, anti-nociceptive and anti-oxidant effects of the aqueous and ethanolic SL extracts. The results of chemical comparison demonstrated that the types of chemicals in the two extracts were identical, but the contents of the main constituents in the aqueous extract were lower than those of the ethanolic extract. As for pharmacological effects, both extracts significantly inhibited inflammatory as well as pain responses in animal models, and also exerted *in vitro* anti-oxidant activities. The inhibitory effects of ethanolic extract were more potent than those of the aqueous extract in all tests. These results suggest that the two preparations should be distinguished when used.

## 2. Results and Discussion

### 2.1. Optimization of Separation Conditions

**Figure 1:**
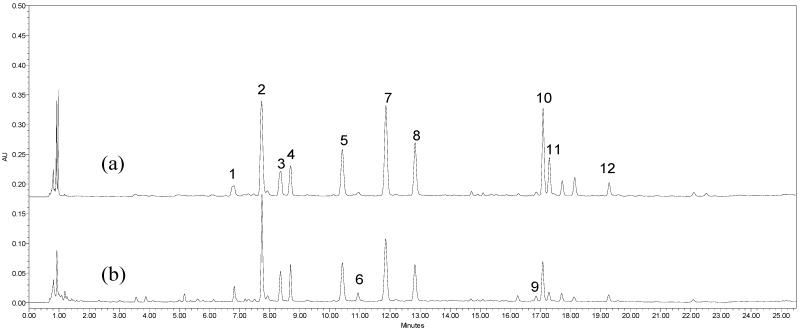
Typical chromatograms of SL ethanolic extract (**a**) and aqueous extract (**b**) at 280 nm.

The conditions for UPLC separation, including type of column, column temperature, detection wavelength and mobile phase gradient, were further optimized based on our previous study. The HSS C_18_ and BEH C_18_ columns were tested at 30, 40 and 50 °C as variables. Mobile phases were screened between acetonitrile and methanol with different gradient elutions. The solvent flow rate was set at 0.3 mL/min according to the instrument manual. Chromatograms of the samples acquired at different wavelengths within 190–500 nm were compared, and peak resolution as well as peak purity were continuously monitored by a PDA detector. After comparison with the BEH C_18_ column, the HSS C_18_ column was chosen as the best separation column because its use resulted in improved separation and superior peak shapes. The results showed that satisfactory separation could be obtained within 25 min by eluting SL extracts on a HSS C_18_ column at 50 °C using a linear gradient of acetonitrile and water. It was found that all of the characteristic peaks of the extracts could be characterized well at a wavelength of 280 nm. Typical UPLC-PDA chromatograms are shown in Figure 1.

### 2.2. Comparison of the Chemical Composition of Two Extracts

Chemical investigation of a herbal medicine is very important because the chemical composition of any material is responsible for its safety and efficacy. Therefore, identification of the main constituents of the two SL extracts became the primary task of the study as a means to track any chemical changes induced by the processing methods.

Based on the *m/z* values obtained in the mass spectra, UV spectra and comparisons with standard compounds, six peaks were unambiguously identified as chlorogenic acid (**2**), syringoside (**3**), 1,3-dicaffeoylquinic acid (**6**), umbelliferone (**7**), scopoletin (**8**) and 1,5-dicaffeoylquinic acid (**10**). By comparing their *m/z* values and UV spectra with the literature data [11,12], five other peaks were tentatively identified as umbelliferone 7-*O*-β-D-glucoside (**1**) and scopoletin 7-*O*-β-D-glucoside (**4**), 3,4-dicaffeoylquinic acid (**9**), 3,5-dicaffeoylquinic acid (**11**) and 4,5-dicaffeoylquinic acid (**12**). The quantitative analysis of the main constituents was performed as previously described [12], and the content of peak 6 was calculated with the calibration curve of peak 10. The results are summarized in Table 1.

**Table 1:** The contents of six constituents in the two SL extracts.

Sample	Contents of the six main constituents in the two extracts (mg/g, *n* = 3)					
	Chlorogenicacid (2)	Syringoside (3)	1,3-Dicaffeoylquinic acid (6)	Umbelliferone (7)	Scopoletin (8)	1,5-Dicaffeoyl quinic acid (10)
Ethanolic extract	24.60 ± 0.74	6.38 ± 0.13	0.45 ± 0.01	23.43 ± 0.45	35.82 ± 0.29	8.54 ± 0.16
Aqueous extract	18.22 ± 0.33	5.41 ± 0.15	0.82 ± 0.02	15.52 ± 0.48	24.20 ± 0.87	3.80 ± 0.13

Values shown are mean ± S.D.

The results demonstrated that the types of chemical composition of the two extracts are identical, namely phenolic acids and coumarins, but the contents of most constituents in the aqueous extract are decreased compared with those in the ethanolic extract. The differences in extraction efficiencies, instability and degradation of the chemical constituents during boiling may contribute to the different contents of chemical constituents in the SL aqueous and ethanolic extracts. On the other hand, the content of 1,3-dicaffeoylquinic acid (peak 6) was increased after by boiling in water. This finding suggests a chemical interaction involving dicaffeoylquinic acids takes place during boiling.

### 2.3. Verification and Elucidation of the Chemical Changes during Boiling

**Figure 2:**
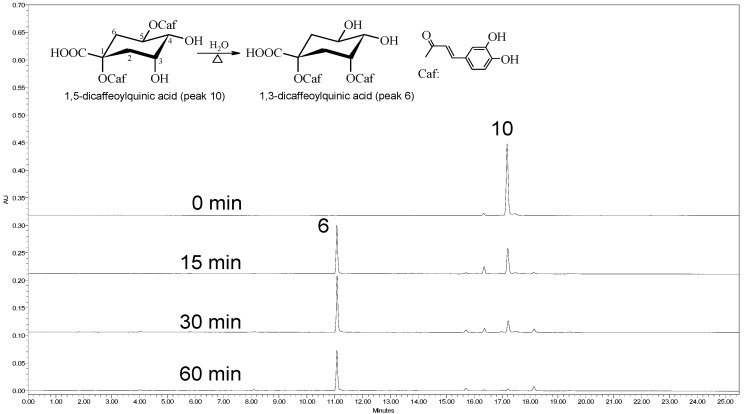
The chromatogram of 1,5-dicaffeoylquinic acid aqueous solution after boiling.

Judging from the above-mentioned identification study, we speculated that the generated 1,3-dicaffeoylquinic acid (peak 6) was derived from 1,5-dicaffeoylquinic acid (peak 10). To verify this hypothesis, we analyzed 1,5-dicaffeoylquinic acid aqueous solution after heating, as a direct approach, without the interference of extraneous material. The results of this analysis are shown in Figure 2.

**Scheme 1:**
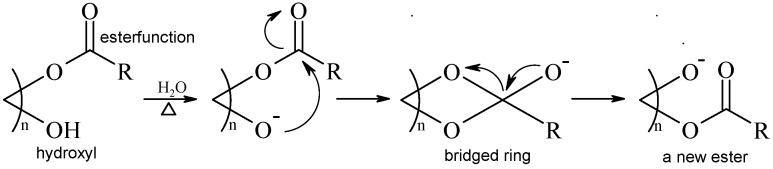
The reaction mechanism of intramolecular trans-esterification.

The results verified that the component 1,3-dicaffeoylquinic acid (peak 6), was a reaction product of 1,5-dicaffeoylquinic acid (peak 10) during heating in water. In addition, we deduced that a transesterification reaction takes place involving the caffeoylquinic acid; a mechanism for this trans formation is shown in Scheme 1.

### 2.4. Comparison of the Anti-Inflammatory Effects of the Two Extracts

SL is traditionally consumed, both as a tincture and as a decoction, to relieve inflammatory and painful disorders [6]. In order to determine whether both processing methods produce extracts of consistent efficacy, it is necessary to compare the anti-inflammatory and anti-nociceptive effects of the two SL extracts.

**Figure 3:**
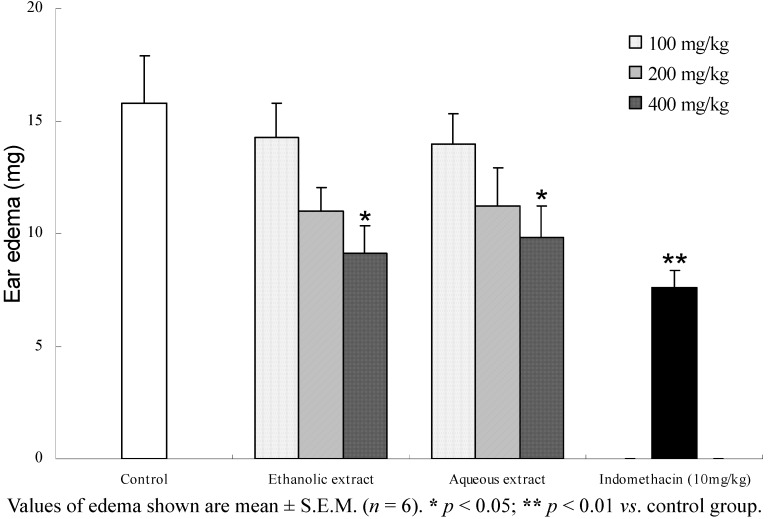
Anti-inflammatory effect of two SL extracts on croton oil-induced ear edema in mice.

In order to measure the anti-inflammatory effects, the present study investigated the depressive effects of the two extracts against croton oil-induced mouse ear edema. A YLS-Q4 punch, which spring-driven sharp blade can reduce the edema fluid loss, was used to remove the ear plugs. Results (Figure 3) show that both SL extracts significantly suppressed the inflammation response at a dose of 100–400 mg/kg in a dose-dependent manner. With the doses of 100, 200 and 400 mg/kg, ethanolic extract exerted a significant inhibition of ear edema by 9.6%, 30.3%, and 42.2%, respectively, and aqueous extract exerted a significant inhibition of ear edema by 11.6%, 28.8%, and 37.8%, respectively. Compared to aqueous extract, ethanolic extract showed higher efficacy against ear edema.

### 2.5. Comparison of the Anti-Nociceptive Effect of Two Extracts

The acetic acid-induced abdominal constriction and hot-plate methods are the animal models typically used to elucidate peripheral and central analgesic activity, respectively [13]. Therefore, the methods of both peripherally and centrally mediated effects were selected for investigating anti-nociceptive effects.

**Table 2:** Anti-nociceptive effects of the two extracts on acetic acid-induced writhing and hot plate test in mice.

Groups	Dose (mg/kg)	Frequency (Counts/15 min)	Inhibition (%)	Latency time (sec)	Increase (%)
Control	-	20.5 ± 1.4	-	14.7 ± 1.8	-
Aqueous extract	100	17.2 ± 1.6	16.1	15.2 ± 2.0	3.4
	200	16.2 ± 2.5	21.0	16.8 ± 1.4	14.3
	400	13.3 ± 1.2 *	35.1	20.7 ± 1.5 *	40.8
Ethanolic extract	100	19.0 ± 2.0	7.3	15.8 ± 1.2	7.5
	200	15.0 ± 1.3	26.8	19.5 ± 1.6	32.7
	400	12.3 ± 1.0 **	40.0	21.7 ± 1.4 *	47.6
Rotundine	50	10.7 ± 1.5 **	47.8	23.0 ± 1.3 **	56.5

Values shown are mean ± S.E.M. (*n* = 6). * *p* < 0.05; ** *p* <0.01 *vs*. control group.

In the acetic acid-induced abdominal writhing test (Table 2), oral administration of SL aqueous extract (100, 200 and 400 mg/kg) resulted in a significant inhibition of writhing by 16.1%, 21.0%, and 35.1%, respectively, and oral administration of SL ethanolic extract (100, 200 and 400 mg/kg) resulted in a significant inhibition of writhings by 7.3%, 26.8%, and 40.0%, respectively. In the hot plate test (Table 2), there was a dose-dependent increase in response to thermal stimulation compared with control mice, when mice were treated with extracts of SL. Oral administration of SL aqueous and ethanolic extract resulted in a significant latency of jumping response by 40.8% and 47.6% when treated at 400 mg/kg, respectively, suggesting that SL has central analgesic properties.

From the animal studies, two findings emerge: firstly, both extracts exhibit the therapeutic efficacies of SL against inflammation and pain. This finding justifies the traditional belief that consuming SL as an herbal medicine can prevent inflammatory and painful diseases; secondly, the anti-inflammatory and anti-nociceptive effects of SL ethanolic extract were more potent than those of the aqueous extract. This implies that discrimination among the two preparations is necessary when using them.

### 2.6. Comparison of the Anti-Oxidant Effect of Two Extracts

Oxidative damage to cells induced by free radicals is one of the important causes of inflammation-related diseases like arthritis, atherosclerosis and even cancer [14]. Free radicals cause oxidative damage to healthy cells and trigger the immune system to produce inflammation; conversely, inflammation creates excess free radicals, which can trigger oxidative stress. Oxidation and inflammation are thus in a vicious bicycle controlled by free radicals, so scavenging free radicals and inhibiting the autocatalytic cascade has become a good strategy for preventative therapy of inflammation-related diseases [15,16].

**Figure 4:**
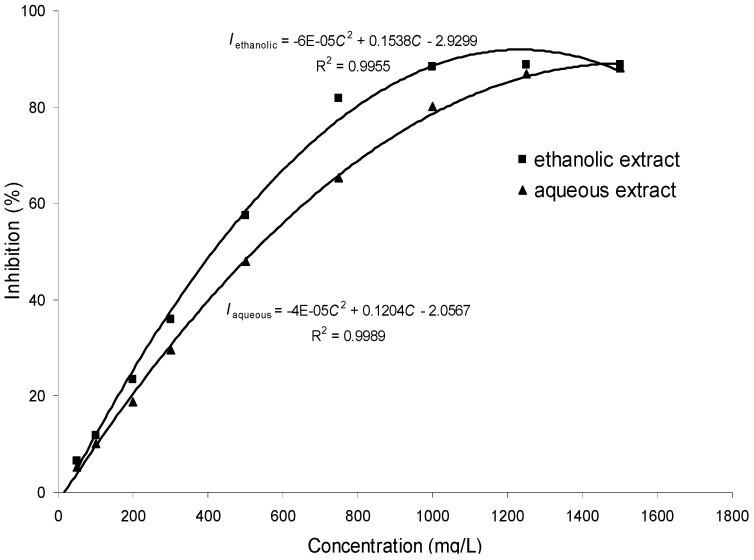
DPPH radical scavenging capacity two SL extracts (*n* = 3).

SL is mainly consumed as an herbal medicine to relieve inflammatory and painful disorders; thus measuring anti-oxidant effects is an accepted approach to evaluate the possible health benefits. In the present study, the anti-oxidant activities of samples were measured by the 2,2-diphenyl-1-picrylhydrazyl (DPPH) radical scavenging assay, and the results, shown in Figure 4, demonstrate the dose-dependent scavenging rate of the two extracts against DPPH radicals. The calibration curve was established by plotting the percent of inhibition against the concentrations of the extracts with nonlinear regression analysis. The nonlinear regression is expressed as *I*_ethanolic_ = −6 × 10^−5^C^2^ + 0.1538C − 2.9299, R^2^ = 0.9955; *I*_aqueous_ = −4 × 10^−5^*C*^2^ + 0.1204*C* − 2.0567, R^2^ = 0.9989, where I and C are the percent of inhibition and the concentration of extract solution, respectively. These regression equations were used for quantifying IC_50_ value of the two extracts; the IC_50_ values were calculated as 409.6 mg/L and 523.4 mg/L for the ethanolic extract and aqueous extract, respectively.

The anti-oxidant study demonstrated that SL might be a free radical scavenger, and both extracts exhibited significant anti-oxidant capacity. Moreover, the ethanolic extract of SL showed higher percentages of oxidant inhibition than the aqueous extract, a finding consistent with the results of the inflammation and pain inhibition tests. The anti-inflammatory effects of botanical anti-oxidants have also been confirmed by other reports, and free radical scavenging is one of the important pathways by which they work [17,18,19]. Based on these findings, we believe that the anti-oxidant capacity of SL probably contributes to its therapeutic efficacy in relieving inflammation and pain by scavenging free radicals.

## 3. Experimental

### 3.1. Materials

The sample of *Saussurea laniceps* (SL) was collected in Tibet (China) in 2008. The identity of the material was confirmed by Dr. Hubiao Chen (School of Chinese Medicine, Hong Kong Baptist University, Hong Kong) on the basis of geographical origin and by means of a macroscopic character assessment. Voucher specimens were deposited in the Chinese Medicines Center, Hong Kong Baptist University.

### 3.2. Preparation of Aqueous and Ethanolic Extracts

Plant materials were cut into small pieces. For the preparation of aqueous extract, SL pieces (ca. 0.2 kg) was boiled in pure water (5 L) for one hour. The supernatant was filtered. This procedure was repeated once, and then the combined filtrates were lyophilized to obtain an aqueous extract. For the preparation of ethanolic extract to represent steeping in spirit, SL sample (0.2 kg) was soaked in 50% ethanol (5 L) at room temperature for 2 days with occasional shaking. The supernatant was filtered. This procedure was repeated once, and then the combined filtrates were evaporated to remove ethanol in a rotary evaporator (40 °C). The wet residues were lyophilized with a Labconco freeze-dry system to obtain an ethanolic extract. About 0.1 g of each extract was dissolved in methanol (25 mL), and the quantitative analysis of the main constituents was performed as previously described [12].

### 3.3. Chemicals and Reagents

Standards of chlorogenic acid, 1,3-dicaffeoylquinic acid and 1,5-dicaffeoylquinic acid were purchased from Biopurity Phytochemcials Ltd. (Chengdu, China). Syringoside and scopoletin were purchased from the National Institute for the Control of Pharmaceutical and Biological Products (Beijing, China). Umbelliferone was purchased from Fluka (Buchs, Switzerland). Croton oil, indomethacin, carboxymethylcellulose and 2,2-diphenyl-1-picrylhydrazyl (DPPH) were purchased from Sigma Chemical Co. (St. Louis, MO, USA). Rotundine was purchased from Zhenang Pharmaceutical Co., Ltd. (Nanjing, China). Ethanol and formic acid of analytical grade were purchased from Merck (Darmstadt, Germany). Acetonitrile of chromatography grade and methanol of analytical grade were purchased from Lab-scan (Bangkok, Thailand). Water was purified using a Milli-Q water system (Millipore; Bedford, MA, USA). Other reagents were analytical grade.

### 3.4. Animals

ICR mice weighing 20–25 g were purchased from the Laboratory Animal Services Center, the Chinese University of Hong Kong. All animals were bred in a temperature-controlled room (23 ± 1 °C) with a 12 h light/dark cycle for a week before the experiment started. Each cage housed six animals and a standard rodent diet and water were provided *ad libitum*. All experimental protocols were approved by the Committee on the Use of Human & Animal Subjects in Teaching and Research of Hong Kong Baptist University, in accordance with the Animals Ordinance (Department of Health, Hong Kong) [20].

### 3.5. Comparison of the Chemical Composition Using UPLC-PDA-ESI/MS

UPLC-PDA-ESI/MS analytical procedures were performed on a Waters Acquity^TM^ ultra-performance liquid chromatography (UPLC) system (Waters Corp., Milford, MA, USA) coupled to a Bruker MicrOTOFQ mass spectrometer by an electrospray ionization (ESI) interface (Bruker Daltonics, Bremen, Germany). The chromatographic separation was performed on a Waters HSS C_18_ column (1.8 μm, 2.1 × 100 mm, Waters Corp.) with a VanGuard^TM^ pre-column (HSS C_18_, 1.7 μm, 2.1 × 5 mm). The column was eluted with a gradient mixture of 0.1% formic acid in water (phase A) and 0.1% formic acid in acetonitrile (Phase B) at the flow rate of 0.3 mL/min. The gradient program was as follows: 3% (B) in 0–2 min, 3–15% (B) in 2–14 min, 15–20% (B) in 14–22 min and 20–30% (B) in 22–25 min. The column temperature was held at 50 °C and the detection wavelength was set to 280 nm. The ESI/MS analysis was conducted in positive ion mode, and the detailed conditions were set by referring to the previous paper [12]. For the chemical comparison, about 0.1 g of each extract was dissolved in 25 mL of methanol, and then filtered through a syringe filter (0.2 μm) to obtain sample solution. To verify the chemical changes during heating, the aqueous solution of 1,5-dicaffeoylquinic acids (50 mg/L) was heated in boiling water bath, and then sampled at intervals after heating. Sample solutions, standard solutions and heated standard solutions (3 μL of each) were injected into the UPLC-PDA-ESI/MS system for analysis.

### 3.6. Comparison of the Anti-Inflammatory Effects Using Croton Oil-Induced Ear Edema in Mice

In the present study, the croton oil-induced ear edema test, a widely accepted model for testing anti-inflammatory effects of substances, was performed with modifications as previously described [21]. The dried extracts were suspended in 1% (w/v) aqueous carboxymethylcellulose for administration to animals. The doses employed are expressed as mg of the dried extract per kg body weight. The administrative dosage for animal experiments was converted from the daily dosage for human beings [6]. An oral administration with vehicle, the SL extracts and indomethacin (10 mg/kg) before inducing ear edema was conducted for 5 consecutive days. Thirty minutes after the last administration of tested extracts, a total of 20 μL of croton oil (2.5%, v/v) in acetone was applied to the surface of the right ear of each mouse. The left ear remained untreated. Control animals received the irritant and an equal volume of aqueous carboxymethylcellulose. The animals were sacrificed by cervical dislocation 4 h later, and the plugs were removed with an YLS-Q4 punch (Yi Yan Technology Development Co., Ltd., Jinan, China) from both the treated and the untreated ear. The difference in weight between the two plugs was taken as a measure of edematous response. Values obtained from experiments were expressed as mean ± S.E.M and further analyzed using one-way ANOVA followed by Dunnett test for multiple comparisons, with the level of significance chosen as *p* < 0.05.

### 3.7. Comparison of the Anti-Nociceptive Effects using Writhing Test and Hot Plate Test in Mice

The writhing test was used as the peripheral pain model [7,22]. Briefly, the extract solution, rotundine (50 mg/kg) and vehicle were orally administered for 5 consecutive days. Half an hour after the last delivery, acetic acid in saline of 0.2 mL (0.7% w/v) was injected intraperitoneally into each animal. The numbers of abdominal writhing movements were counted for 15 min starting 5 min after injection. The hot plate test was used as the thermal pain model [7,23]. Briefly, the extracts solution, rotundine (50 mg/kg, reference drug) and vehicle were orally administered for 5 consecutive days. Half hour after the last delivery, each mouse was placed on a hot-plate surface (IITC Model 39, Woodland Park, CA) maintained at 55 ± 0.2 °C. The reaction time from hot-plate placement to hind-paw lick was recorded. Values obtained from experiments were expressed as mean ± S.E.M and further analyzed using one-way ANOVA followed by Dunnett test for multiple comparisons, with the level of significance chosen as *p* < 0.05.

### 3.8. Comparison of the Anti-Oxidant Effects Using DPPH Test

Experiments were carried out according to the previous report with modifications [24,25,26]. Briefly, DPPH radicals were prepared in ethanol at a concentration of 0.08 mM. The two extracts were dissolved in water and further diluted to various concentrations for assay. A fixed amount (0.2 mL) of the test solutions was added to DPPH solution (1.8 mL) and vortexed thoroughly. After incubation in the dark at room temperature for 30 min, the absorbance of the mixture was measured utilizing a UV/Vis spectrophotometer (Jasco V-530) at 517 nm against a blank of ethanol. Water was used in the control test, while chlorogenic acid served as the reference drug. The anti-oxidant effect of samples is expressed as percent of inhibition (I) using the following equation:


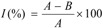
(1)

where A is the absorbance of control reaction (water with DPPH) and B is the absorbance of the tested sample reaction (sample with DPPH). All tests were run in triplicate, and values obtained from experiments were averaged.

## 4. Conclusions

To understand the effects of processing on the composition and efficacy of SL herbal extract, the present study is the first to compare the chemical composition and pharmacological effects of aqueous and ethanolic extracts of SL prepared by boiling in water and steeping in spirit containing 50% ethanol, respectively. The chemical study results demonstrated that the types of chemical constituents are identical in aqueous extract and ethanolic extract, while the contents of the main constituents in the ethanolic extract were higher than those of the aqueous extract. Compared with the processing by steeping in spirit, a transesterification of 1,5-dicaffeoylquinic acids takes place during boiling, generating 1,3-dicaffeoylquinic acid. The results of the *in vivo* anti-inflammatory and anti-nociceptivetests verified the therapeutic efficacy of SL as an herbal medicine for relieving inflammation and pain, and the ethanolic extract exhibited more potency than the aqueous extract. Moreover, the result of *in vitro* anti-oxidant test also highlighted that SL serves as a free radical scavenger, and the therapeutic effects of SL may be correlated with its anti-oxidant capacity. This study provides evidence that SL is a beneficial herbal medicine for relieving inflammation and pain, whether used as an aqueous or ethanolic extract. This study also suggests that the two preparations should be distinguished when used, due to their different therapeutic potencies.
